# Excited-State Dynamics of Proflavine after Intercalation into DNA Duplex

**DOI:** 10.3390/molecules27238157

**Published:** 2022-11-23

**Authors:** Jie Zhou, Yanyan Jia, Xueli Wang, Menghui Jia, Haifeng Pan, Zhenrong Sun, Jinquan Chen

**Affiliations:** 1 State Key Laboratory of Precision Spectroscopy, East China Normal University, Shanghai 200241, China; 2 Key Laboratory for Advanced Materials, Feringa Nobel Prize Scientist Joint Research Center, Institute of Fine Chemicals, School of Chemistry & Molecular Engineering, East China University of Science and Technology, Shanghai 200237, China; 3 Collaborative Innovation Center of Extreme Optics, Shanxi University, Taiyuan 030006, China

**Keywords:** excited-state dynamics, proflavine, DNA duplex, femtosecond transient-absorption, fluorescence-quenching

## Abstract

Proflavine is an acridine derivative which was discovered as one of the earliest antibacterial agents, and it has been proven to have potential application to fields such as chemotherapy, photobiology and solar-energy conversion. In particular, it is well known that proflavine can bind to DNA with different modes, and this may open addition photochemical-reaction channels in DNA. Herein, the excited-state dynamics of proflavine after intercalation into DNA duplex is studied using femtosecond time-resolved spectroscopy, and compared with that in solution. It is demonstrated that both fluorescence and the triplet excited-state generation of proflavine were quenched after intercalation into DNA, due to ultrafast non-radiative channels. A static-quenching mechanism was identified for the proflavine-DNA complex, in line with the spectroscopy data, and the excited-state deactivation mechanism was proposed.

## 1. Introduction

Proflavine (3,6-diaminoacridine) is a planar aromatic molecule and it has the ability to intercalate into base pairs in double-stranded DNA [1]. Because of this, it could interfere with many crucial biological functions [2]. It has been shown that proflavine as well as its derivatives can lead to mutations in viruses and bacteria and DNA-breaking in cells [3,4,5,6], possibly due to the ability of proflavine to stabilize DNA-topoisomerase II intermediates [7,8,9,10,11]. Meanwhile, proflavine, as well as its derivatives, is highly photosensitive, due to its acridine scaffold [12]. Therefore, the interactions between proflavine and other agents were extensively studied, using spectroscopic techniques [13,14,15,16]. For example, proflavine exhibits excited-state proton-transfer properties when it is encapsulated in a silane-modified MCM-41 silicate host, as well as in amine-functionalized MCM-41 [17,18]. Proflavine can also access triplet excited-states [19] and be used as a photosensitizer in sensitization experiments [20], or generate free radicals to participate in the photoreaction [6] after excitation. Moreover, it has been used as a photocatalyst to promote the reduction of aldehyde hydrides induced by visible light [19].

Meanwhile, as a DNA intercalating agent, proflavine has been used to probe the dynamics of DNA [21,22,23]. When proflavine is inserted into A-T base pairs, its fluorescence is slightly enhanced. On the other hand, fluorescence intensity decrease significantly when proflavine is inserted into G-C base pairs [24]. It is known that the environment around DNA has a great influence on the effect of proflavine intercalation. The intercalation efficiency will be reduced when the sodium-ions concentration is high in solution [25] or when hydrogen bonding between water and DNA is inhibited [26]. When proflavine binds to double-stranded DNA, the absorption of proflavine will be red shifted, he peak intensity will decrease, and the relative viscosity of DNA will also increase [13]. Moreover, proflavine can also promote short oligonucleotides to form paired long chains [27]. It is also shown to inhibit thymidine-dimer formation [28] and promote the division of dimers that have already formed [29].

However, the interaction between proflavine and DNA has not been studied from the aspect of excited-state dynamics in detail. In 2012, Kumar and co-workers studied the fluorescence kinetics of proflavine in different solvents, and proposed that proflavine has one emission state at 500 nm with 50 ps lifetime, as well as another state that emits at 525 nm with 4.8 ns lifetime [30]. Later, the same group studied the change of proflavine-fluorescence lifetime after intercalation into G-quadruplex DNA [31]. They found that fluorescence lifetime is shortened to 800 ps. As mentioned above, the fluorescence of proflavine is quenched after intercalation into G-C base pairs, but the detailed quenching mechanism remains unknown. Herein, we study the excited-state dynamics of proflavine after intercalation into d(GC)_9_ DNA duplex in detail, using both steady-state and femtosecond time-resolved spectroscopy. The fluorescence-quenching mechanism of proflavine into DNA duplex and the excited-state deactivation mechanism were proposed, based on the spectroscopy data.

## 2. Materials and Methods

**Sample preparation.** Proflavine hydrochloride was purchased from Sigma–Aldrich, and DNA duplex d(GC)_9_ from Shanghai Generay Biotech Co., Ltd. (Shanghai, China). All materials were used as received, without further purification. Proflavine hydrochloride and d(GC)_9_ were dissolved in the phosphate buffer solution (PBS, pH = 7.4) and stored in the dark. The PBS solution was prepared using 50 mM monosodium phosphate and disodium phosphate salts solution and 100 mM sodium chloride solution. All the water used in the experiment was 18.2 MΩ deionized water (Direct-Q3 UV, Merck Millipore). We dissolved the purchased d(GC)_9_ single strand in the configured PBS, annealed the solution at 95 °C for 10 min, and then slowly cooled it to room temperature within 30 min prior to the TA experiments, to finally form B-type DNA d(GC)_9_d(GC)_9_ double strand at a concentration of 50 μM.

**UV/Vis Absorption and fluorescence emission spectroscopy.** UV–vis absorption spectra were measured using a dual beam UV–vis spectrometer (TU1901, Purkinje General Instrument Co. Ltd., Beijing China). Fluorescence emission spectra were obtained by using a steady-state fluorescence spectrometer (FluoroMax-4 spectrofluorometer, Horiba, Jobin Yvon).

**Fluorescence quenching constant.** There are three main types of fluorescence quenching: dynamic quenching, static quenching, and quenching combined with both, which can be determined by the Stern–Volmer equation below [32]:

$$\frac{F_{0}}{F}=K_{SV}\left[Q\right]+1=K_{q}\tau_{0}\left[Q\right]+1,$$

$F_{0}$ is the initial fluorescence-intensity, F is the fluorescence intensity varying with the concentration of the quencher, $K_{SV}$ is the fluorescence quenching constant, [Q] is the concentration of the quencher, in this work, the quenching agent is d(GC)_9_, $K_{q}$ represents the quenching rate-constant, $\tau_{0}$ is the lifetime of the fluorophore without quencher.

**Binding constant.** There are many binding sites between small molecules and biological macromolecules. The formula for calculating the number of binding sites is as follows [32]:

$$log\frac{F_{0}-F}{F}=logK_{a}+nlog\left[Q\right],$$

$F_{0}$ is the initial fluorescence-intensity, F is the fluorescence intensity varying with the concentration of the quencher, $K_{a}$ is the binding constant, n is the number of binding sites, [Q] is the concentration of the quencher. In this work, the quenching agent is d(GC)_9_. The calculated binding-site for proflavine in DNA duplex at room temperature is 0.77. Therefore, when making proflavine-DNA, we keep the concentration ratio of proflavine and DNA duplex in the solvent at 0.75, to ensure that proflavine is inserted into DNA duplex as much as possible.

**Fluorescence lifetime.** Fluorescence lifetime was measured using a home-built time-correlated single-photon counting (TCSPC) system. The picosecond super-continuum fiber laser (SC400-pp-4, Fianium, Southampton, UK) provides excitation pulse with a repetition rate of 20 MHz, and fluorescence was recorded on the TCSPC module (PicoHarp 300, PicoQuant, Berlin, Germany) and a microchannel plate-PMT (R3809U-50, Hamamatsu, Shizuoka, Japan). A monochromator (7ISW151, Sofn Instruments, Beijing, China) was used to select the emission wavelength. The instrument response function (IRF) of the system was determined to be ~200 ps, by measuring the scattering of silica solutions.

**Femtosecond broadband transient-absorption (TA).** Femtosecond broadband TA experiments were carried out using a transient-absorption spectrometer (Helios-Eos Fire, Ultrafast System) [33,34,35]. All experiments were performed at room temperature and a 2 mm fused-silica cuvette was used in the TA experiments. A Ti: sapphire laser system (Astrella, 800 nm, 90 fs, 7 mJ per pulse and 1 kHz repetition rate, Coherent Inc., Santa Clara, CA, USA) generated the fundamental beam. One part was used to generate a 440 nm pump beam used in the experiment, through an optical parametric amplifier (OPerA Solo, Coherent In.). Another part passed through the calcium-fluoride window to generate a white-light continuum (WLC) probe beam. The polarization between pump and probe beams was set to be the magic angle (54.7°). The instrument response function (IRF) of the TA system was ~120 fs, measuring the solvent response under the same experimental conditions. Nanosecond TA spectra were measured using a TA spectrometer (Helios-EOS fire, Ultrafast System). The excitation beam was derived from the same Ti:sapphire amplifier as the femtosecond experiments, and the probe light (supercontinuous white light) in the optical path comes from the electronic trigger-diode, which is connected to the laser with an electronic switch, to realize the time resolution. In this system, the probe light is divided into signal beam and reference beam, to improve the signal-to-noise ratio of the system. The IRF is 100 ps and the detection range is 360–900 nm.

## 3. Results and Discussion

**Figure 1:**
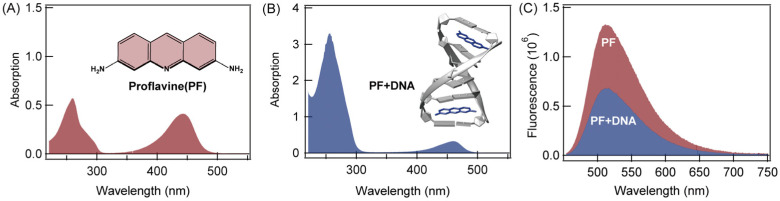
(**A**) The absorption spectra of proflavine in buffer solution (pH = 7.4) and the chemical structure of proflavine; (**B**) the absorption spectra and structure of the intercalation complex of proflavine-DNA (PF-DNA) in buffer solution (pH = 7.4); (**C**) fluorescence emission spectra of 5 μM proflavine and fluorescence emission spectra of 5 μM proflavine with 2.5 μM DNA in buffered solution (pH = 7.4).

Figure 1A shows the absorption spectra of proflavine (PF) in buffer solution (pH = 7.4) and the structure of proflavine. Proflavine has two absorption peaks, and the maximum are at 260 nm and 444 nm, respectively. Figure 1B shows the absorption spectra and structure of the intercalation complex of PF-DNA in buffer solution (pH = 7.4). The complex also has two absorption peaks. The absorption peak (255 nm) in the ultraviolet region is the combination of the absorbance of both proflavine and DNA, and the peak in the visible region (460 nm) is mainly composed of proflavine itself, after intercalation into the DNA duplex. At the same concentration of proflavine, the main peak of PF-DNA in the visible region shows a 16 nm red-shift, and the amplitude also decreases by 21%, which is a typical spectroscopy feature indicating intercalation of proflavine into DNA duplex [13]. Figure 1C compares the fluorescence emission spectra of 5 μM proflavine in PBS solution (pH = 7.4) with that of 5 μM proflavine with 2.5 μM DNA in solution. It is clear that the fluorescence of proflavine is quenched after intercalation into DNA duplex. The fluorescence quantum-yield of PF is 38.56%, and that of PF-DNA is 2.86%.

**Figure 2:**
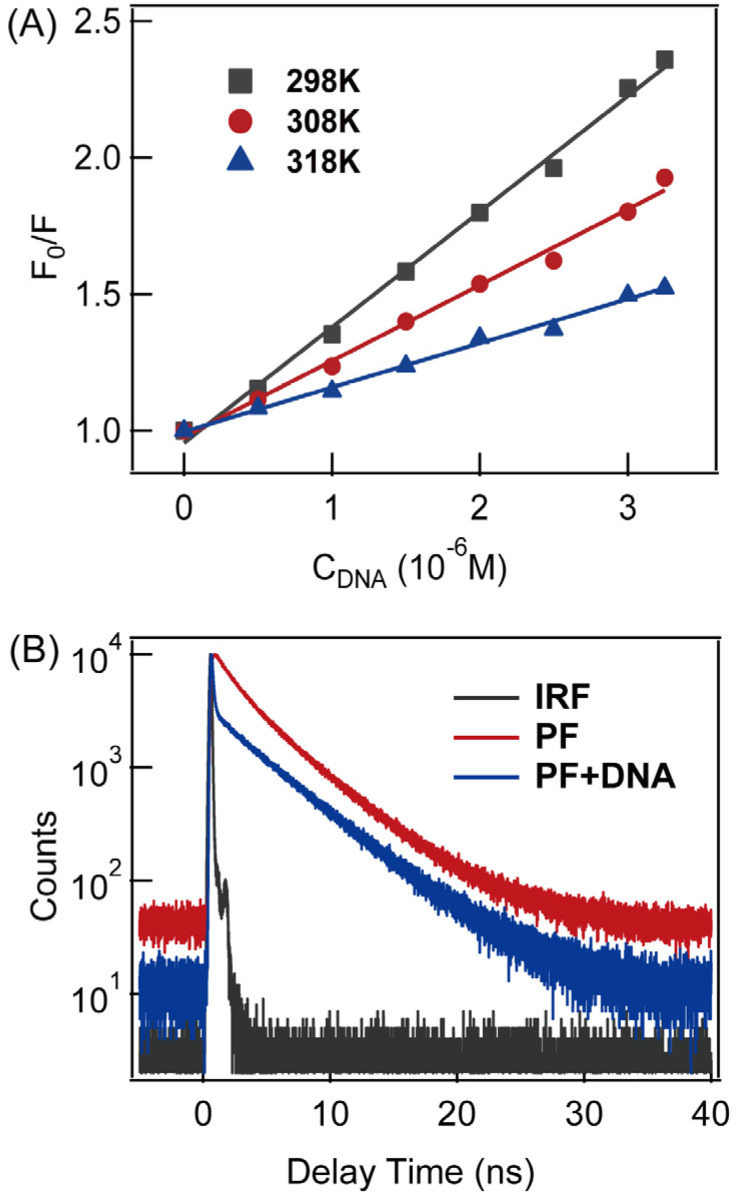
(**A**) Fluorescence quenching experiments of proflavine (5 μM) at indicated temperatures in buffer solution (pH = 7.4); the fluorescence quenching constant ($K_{SV}$) was determined to be (4.2 ± 0.1) × 10^5^ M^−1^, (2.8 ± 0.1) × 10^5^ M^−1^ and (1.6 ± 0.1) × 10^5^ M^−1^ at 298 K, 308 K and 318 K, respectively; (**B**) fluorescence kinetics of proflavine (15 μM) and PF-DNA (15 μM PF with 20 μM DNA) in buffer solution (pH = 7.4); IRF is the instrument-response function of TCSPC system.

In order to clarify the fluorescence-quenching mechanism, temperature-dependent quenching experiments were carried out, and the results were displayed in Figure 2A. Linear correlations were found at different temperatures. The quenching rate-constant ($K_{q}=K_{SV}/\tau_{0}$) values at all temperature studied are at the level of 10^13^ M^−1^·s^−1^, which is much larger than the usual definition of static quenching (>10^10^ M^−1^·s^−1^) [32]. Therefore, the quenching mechanism of PF in DNA should be static quenching. To verify such a hypothesis, fluorescence lifetime was measured for both proflavine and PF-DNA. As shown in Figure 2B, a 4.6 ± 0.2 ns lifetime was found for proflavine itself, while there is an additional ultrafast-fluorescence decay component (<200 ps, instrumental response-time), which appeared after proflavine was intercalated into DNA duplex. This result is in line with a previous report stating that ultrafast-fluorescence decay was seen when proflavine was inserted into G-quadruplex DNA [31], in which an ultrafast-fluorescence component was found. Therefore, we believe that the fluorescence-quenching mechanism of proflavine in DNA should belong to static quenching.

**Figure 3:**
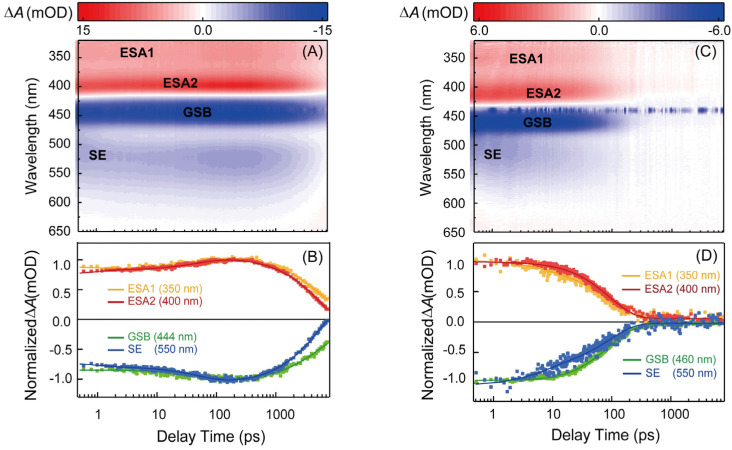
(**A**) Femtosecond time-resolved transient-absorption spectra and (**B**) representative kinetic traces extracted from global fitting of transient-absorption data of proflavine (50 μM) in buffer solution (pH = 7.4); (**C**) femtosecond time-resolved transient-absorption spectra and (**D**) representative kinetic traces extracted from global fitting of transient-absorption data of the intercalation complex of PF−DNA (50 μM) in buffer solution (pH = 7.4).

Femtosecond broadband transient-absorption (TA) experiments were conducted to further study the ultrafast excited-state dynamics of PF-DNA. Figure 3A,B show the TA spectra and kinetics of the proflavine in PBS (pH = 7.4) under 440 nm excitation. There are two positive excited-state absorption (ESA) bands in the spectra, and the peaks were seen at 350 nm and 400 nm, respectively. The negative TA signal with peaks at 444 nm and 512 nm in the spectra correspond to the absorption and fluorescence of the proflavine itself, and they are marked as ground state bleaching (GSB) signal and stimulated emission (SE) signal in Figure 3A. From the TA spectra and kinetics, we can see that proflavine has a long-lived component. Figure S1 exhibits the nanosecond TA spectra. The two lifetimes were determined to be 4.7 ns and 1.3 μs. It is clear that the lifetime of the 1.3 μs component is longer under the N_2_-saturated condition, as shown in Figure S2, indicating that this component must arise from the triplet state of proflavine, as reported in the literature [19].

**Figure 4:**
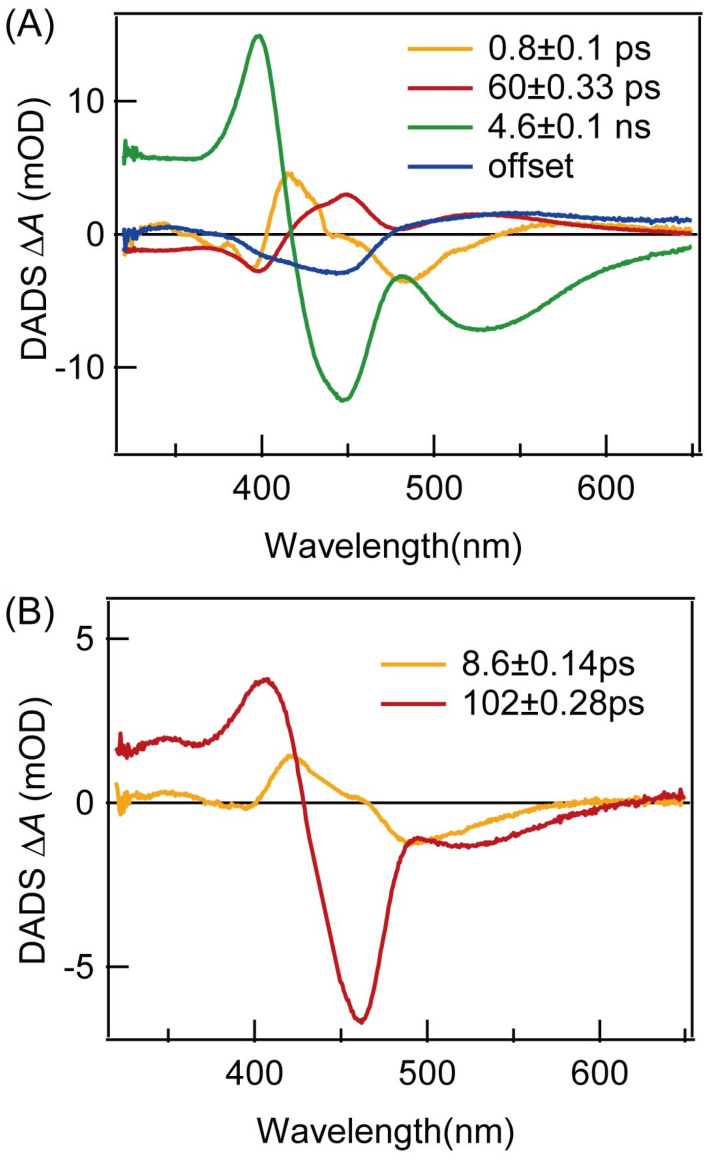
Decay-associated difference spectra (DADS) extracted from the global fitting of TA data for (**A**) proflavine and (**B**) PF−DNA in buffer solution (pH = 7.4).

Global analysis of the TA spectra of proflavine itself yielded four lifetimes, and the decay-associated difference spectra (DADS) are shown in Figure 4A. The first DADS (orange) has a lifetime of 0.8 ps and it has negative amplitude around the 400 nm and 450–540 nm region, suggesting that the 0.8 ps component could decay to both the 60 ps and 4.6 ns components, simultaneously. The second DADS (red) has a lifetime of 60 ps and it has positive amplitude around the 415–600 nm region, but a dip around 490 nm. There is no GSB signal in the first two DADS, suggesting they do not contribute to the ground-state repopulation (Figure S3). The third DADS (green) has a lifetime of 4.6 ns and it has positive amplitude around the 320–416 nm region and negative amplitude around the 416–650 nm region. This DADS is similar to the whole TA spectra, and 480–560 nm is the stimulated-emission signal of proflavine. The last DADS (blue) has a long lifetime, and it is assigned to a triplet state, based on the ns TA experiments above. Previously, fluorescence up-conversion experiments were used to demonstrate that proflavine has three lifetimes in water [30]. The 1.36 ps lifetime is assigned to solvent relaxation, while the 71.58 ps and 4.6 ns lifetimes are assigned to the S1 (ππ*) and S2 (ππ*) state, respectively. The lifetimes determined in our TA experiments on proflavine are similar to those in the above study, and we believe that the 0.8 ps lifetime is solvent relaxation and that the 60 ps and 4.6 ns lifetimes correspond to the lifetimes of the S1 (ππ*) and S2 (ππ*) state, respectively.

Figure 3C,D show the TA spectra and kinetics of the PF-DNA in PBS (pH = 7.4), under 440 nm excitation. When proflavine is intercalated into the DNA duplex, the TA spectra are similar in shape to proflavine itself. However, the lifetimes are much shorter. Moreover, the triplet state is completely quenched as the GSB signal returns to base line within ~500 ps after excitation. Global fitting yielded only two lifetimes, and DADS are shown in Figure 4B. The first DADS (orange) now has a lifetime of 8.6 ps and it has negative amplitude around the 400 nm and 450–540 nm region. The peak of the negative amplitude is located at 500 nm, which is consistent with the luminescence wavelength of the proflavine S1 state. The second DADS (red) has a lifetime of 102 ps and it has positive amplitude around the 320–430 nm region, while there is negative amplitude around the 430–650 nm region. The negative amplitude includes the GSB signal around the 430–490 nm region and the SE signal around the 490–650 nm region. In the SE signal, the peak is located at 525 nm, which is consistent with the fluorescence wavelength of the S2 state of proflavine. Therefore, it is clear that both emissive states in proflavine are quenched after intercalation into DNA duplex, as the lifetimes are one order of magnitude smaller. It has been suggested that a charge-transfer state could form between the guanine base and proflavine, and this can lead to a red-shift in the absorption spectra of proflavine, together with a weak blue-shift of the fluorescence spectra [31,36]. In our experiments, the changes in absorption and emission spectra are in line with the above studies. Therefore, it is possible that a charge-transfer state can form in PF-DNA, and the main relaxation pathway is charge recombination rather than fluorescence emission. However, the typical G∙+ radical signal was not clearly resolved in our TA spectra, possibly due to overlapping with the TA spectra of PF-DNA. On the other hand, it is shown that the environmental polarity around the proflavine molecule will change when proflavine is inserted into DNA [30]. This could reduce the energy level of proflavine and lead to a red-shift in the absorption spectra. In this case, it may also introduce non-radiative pathways in PF-DNA, which can quench the fluorescence emission. Further experiments and calculations are necessary to verify these two hypotheses. Nevertheless, our TA results can serve as a benchmark for further studies.

Finally, it is worth pointing out that the triplet state of proflavine is completely quenched in PF-DNA. Indeed, proflavine can react with oxygen to form peroxide radicals to participate in light reactions [6], and can also be used as a photosensitizer in sensitization experiments [20]. Because there is no triplet state and free radical in PF-DNA, it should be a stable molecule after excitation. Therefore, the reported physiological effect of proflavine is not an excited-state problem [31].

## 4. Conclusions

**Scheme 1:**
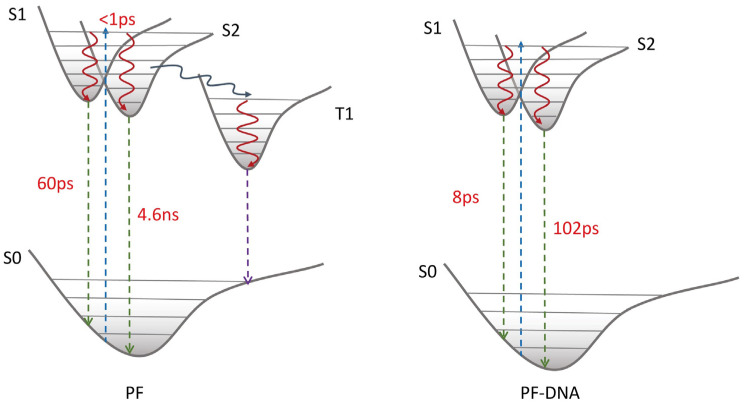
Excited-state relaxation mechanisms of PF and PF-DNA.

In this paper, we studied the excited-state dynamics of proflavine and PF-DNA, in detail. With the help of both steady-state and time-resolved spectroscopy, the fluorescence quenching in PF-DNA is found to be a static-quenching mechanism. Both emissive states in proflavine exhibit one-order-of-magnitude smaller excited-state lifetimes in PF-DNA. Moreover, the triplet state of proflavine is also quenched in PF-DNA. The full picture of the excited-state relaxation mechanism of proflavine and PF-DNA is proposed and shown in Scheme 1. After the proflavine monomer is excited, it can relax into both S1 and S2 states rapidly from the Franck–Condon region, and then these two states can decay back to the ground state with lifetimes of 60 ps and 4.6 ns, respectively. At the same time, proflavine could also undergo intersystem crossing to a long-lived triplet state. When the proflavine is inserted into the DNA duplex, the excited-state relaxation mechanism is different from the monomer. After being excited, PF-DNA shows effective non-radiation decay to the ground state, and the lifetimes of S1 and S2 states are shortened into 8 ps and 102 ps, respectively. These results provide new insights into understanding the interaction between proflavine and DNA, and could provide benchmark information for future experimental and computational studies on proflavine derivatives in the biological environment.

## Data Availability

Data will be made available on request.

## Supplementary Materials

The following supporting information can be downloaded at: https://www.mdpi.com/article/10.3390/molecules27238157/s1, Figure S1: nanosecond TA spectra and lifetime of proflavine; Figure S2: comparison of proflavine lifetime before and after deoxygenation.

## Sample Availability

Samples of the compounds are available from the authors.

## References

[1] Jin B., Sung G.W., Jang Y.J. (2019). Binding Mode of Proflavine to DNA Probed by Polarized Light Spectroscopy. J. Chin. Chem. Soc..

[2] Kožurková M., Sabolová D., Kristian P. (2017). A Review on Acridinylthioureas and Its Derivatives: Biological and Cytotoxic Activity. J. Appl. Toxicol..

[3] Calberg-Bacq C.M., Siquet-Descans F., Piette J., Van de Vorst A. (1977). Free Radical Induction in Bacteriophage øX174 DNA after Exposure to Proflavine and Visible Light. Biochim. Biophys. Acta BBA Nucleic Acids Protein Synth..

[4] Piette J., Lopez M., Bacq C.M.C., Van de Vorst A. (1981). Mechanism for Strand-Break Induction in DNA-Proflavine Complexes Exposed to Visible Light. Int. J. Radiat. Biol. Relat. Stud. Phys. Chem. Med..

[5] Piette J., Decuyper J., Machiroux R., Calberg-Bacq C.M., Van de Vorst A., Lion Y. (1982). Visible-Light-Induced OH Radicals in DNA-Proflavine Complexes: An e.p.r. and Spin Trapping Study. Int. J. Radiat. Biol. Relat. Stud. Phys. Chem. Med..

[6] Gatasheh M.K., Kannan S., Hemalatha K., Imrana N. (2017). Proflavine an Acridine DNA Intercalating Agent and Strong Antimicrobial Possessing Potential Properties of Carcinogen. Karbala Int. J. Mod. Sci..

[7] Ferguson L.R., Denny W.A. (1991). The Genetic Toxicology of Acridines. Mutatation Res..

[8] Tsankov N., Kazandjieva J., Drenovska K. (1998). Drugs in Exacerbation and Provocation of Psoriasis. Clin. Dermatol..

[9] Demeunynck M. (2004). Antitumor Acridines. Expert Opin. Ther. Pat..

[10] Goodell J.R., Madhok A.A., Hiasa H., Ferguson D.M. (2006). Synthesis and Evaluation of Acridine- and Acridone-Based Anti-Herpes Agents with Topoisomerase Activity. Bioorgan. Med. Chem..

[11] Tonelli M., Vettoretti G., Tasso B., Novelli F., Boido V., Sparatore F., Busonera B., Ouhtit A., Farci P., Blois S. (2011). Acridine Derivatives as Anti-BVDV Agents. Antivir. Res..

[12] Lerman L.S. (1961). Structural Considerations in the Interaction of DNA and Acridines. J. Mol. Biol..

[13] Aslanoglu M. (2006). Electrochemical and Spectroscopic Studies of the Interaction of Proflavine with DNA. Anal. Sci..

[14] Weinstein I.B., Finkelstein I.H. (1967). Proflavine Inhibition of Protein Synthesis. J. Biol. Chem..

[15] Qu X., Chaires J. (2000). Analysis of Drug-DNA Binding Data. Methods Enzymol..

[16] Prasher P., Sharma M. (2018). Medicinal Chemistry of Acridine and Its Analogues. Medchemcomm.

[17] Ananthanarayanan K., Selvaraju C., Natarajan P. (2007). Novel Excited State Proton Transfer Reaction Observed for Proflavine Encapsulated in the Channels of Modified MCM-41. Microporous Mesoporous Mater..

[18] Ananthanarayanan K., Natarajan P. (2009). Fabrication and Photophysical Studies of Phenosafranine and Proflavine Dyes Encapsulated in Mesoporous MCM-41 along with Titanium Dioxide Nanoparticles. Microporous Mesoporous Mater..

[19] Ghosh T., Slanina T., Konig B. (2015). Visible Light Photocatalytic Reduction of Aldehydes by Rh(iii)-H: A Detailed Mechanistic Study. Chem. Sci..

[20] Tokumura K., Matsushita Y. (2001). Triplet-Sensitized Deoxygenation Reaction of 6-Cyanophenanthridine 5-Oxide in Ethanol. J. Photochem. Photobiol. A Chem..

[21] Strauss G., Broyde S.B., Kurucsev T. (1971). Concentration Quenching of Proflavine Hydrochloride in Dry Films of Sodium Deoxyribonucleate and Poly(Vinyl Alcohol). J. Phys. Chem..

[22] Ramstein J., Ehrenberg M., Rigler R. (1980). Fluorescence Relaxation of Proflavin-Deoxyribonucleic acid Interaction. Kinetic Properties of a Base-Specific Reaction. Biochemistry.

[23] Ricci C.G., Netz P.A. (2009). Docking Studies on DNA-Ligand Interactions:Building and Application of a Protocol to Identify the Binding Mode. J. Chem. Inf. Model..

[24] Disteche C., Bontemps J., Houssier C., Frederic J., Fredericq E. (1980). Quantitative Analysis of Fluorescence Profiles of Chromosomes. Influence of DNA Base Composition on Banding. Exp. Cell Res..

[25] Jain S.S., LaFratta C.N., Medina A., Pelse I. (2013). Proflavine–DNA Binding Using a Handheld Fluorescence Spectrometer: A Laboratory for Introductory Chemistry. J. Chem. Educ..

[26] Qu X., Chaires J.B. (2001). Hydration Changes for DNA Intercalation Reactions. J. Am. Chem. Soc..

[27] Jain S.S., Anet F.A., Stahle C.J., Hud N.V. (2004). Enzymatic Behavior by Intercalating Molecules in a Template-Directed Ligation Reaction. Angew. Chem. Int. Ed..

[28] Sutherl B.M., Sutherland J.C. (1969). Mechanisms of Inhibition of Pyrimidine Dimer Formation in Deoxyribonucleic Acid by Acridine Dyes. Biophys. J..

[29] Setlow R.B., Carrier W.L. (1967). Formation and Destruction of Pyrimidine Dimers in Polynucleotides by Ultra-Violet Irradiation in the Presence of Proflavine. Nature.

[30] Kumar K.S., Selvaraju C., Malar E.J., Natarajan P. (2012). Existence of a New Emitting Singlet State of Proflavine: Femtosecond Dynamics of the Excited State Processes and Quantum Chemical Studies in Different Solvents. J. Phys. Chem. A.

[31] Kumar V., Sengupta A., Gavvala K., Koninti R.K., Hazra P. (2014). Spectroscopic and Thermodynamic Insights into the Interaction between Proflavine and Human Telomeric G-Quadruplex DNA. J. Phys. Chem. B.

[32] Xiong Y., Li J., Huang G., Yan L., Ma J. (2021). Interacting Mechanism of Benzo(a)pyrene with Free DNA In Vitro. Int. J. Biol. Macromol..

[33] Liu Y.-Y., Pan H.-F., Xu J.-H., Chen J.-Q. (2021). Long Chain Fatty Acid Affects Excited State Branching in Bilirubin-Human Serum Protein Complex. Chin. J. Chem. Phys..

[34] Chen Z., Liu Y.-Y., He X.-X., Chen J.-Q. (2020). Ultrafast Excited State Dynamics of Biliverdin Dimethyl Ester Coordinate with Zinc Ions. Chin. J. Chem. Phys..

[35] Li X., Wang X., Lv M., Zhou Z., Pan H., Chen J. (2022). Direct Observation of a Singlet ππ* and nπ* Equilibrium State in 2-Amino-1,3,5-Trainzine Solution. Chin. J. Chem. Phys..

[36] MoradpourHafshejani S., Hedley J.H., Haigh A.O., Pike A.R., Tuite E.M. (2013). Synthesis and Binding of Proflavine Diazides as Functional Intercalators for Directed Assembly on DNA. RSC Adv..

