# Supplementary material for: Excited-State Dynamics of Proflavine after Intercalation into DNA Duplex
Source: Molecules. 2022 Nov 23;27(23):8157. doi: 10.3390/molecules27238157 (PMC9738913; doi:10.3390/molecules27238157)
Supplement: Supplementary file 1 [file molecules-27-08157-s001.zip › molecules-2028273-supplementary.pdf]

Supporting Materials

Excited-State Dynamics of Proflavine after Intercalation into DNA Duplex

Jie Zhou [1], Yanyan Jia [2,*], Xueli Wang [1], Menghui Jia [1], Haifeng Pan [1], Zhenrong Sun [1] and Jinquan Chen [1,3*]

[1] State Key Laboratory of Precision Spectroscopy, East China Normal University, Shanghai 200241, China; 51200920043@stu.ecnu.edu.cn (J.Z.); xlwang@lps.ecnu.edu.cn (X.W.); mhjia@lps.ecnu.edu.cn (M.J.); hfpan@phy.ecnu.edu.cn (H.P.); zrsun@phy.ecnu.edu.cn (Z.S.)

[2] Key Laboratory for Advanced Materials and Feringa Nobel Prize Scientist Joint Research Center,
Institute of Fine Chemicals, School of Chemistry & Molecular Engineering, East China University of Science and Technology, Shanghai 200237, China

[3] Collaborative Innovation Center of Extreme Optics, Shanxi University, Taiyuan 030006, China

* Correspondence: jiayanyan@ecust.edu.cn (Y.J.); jqchen@lps.ecnu.edu.cn (J.C.)

Additional TA Results

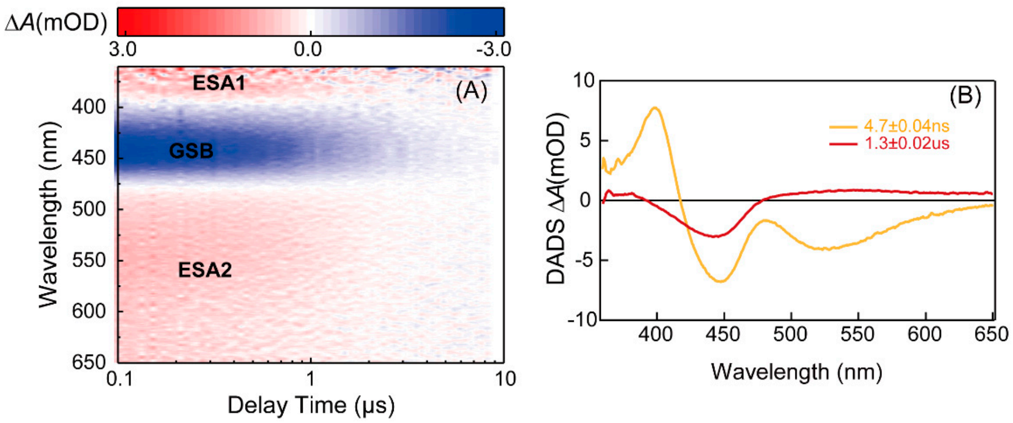

Figure S1. Nanosecond TA spectra and lifetime of proflavine.

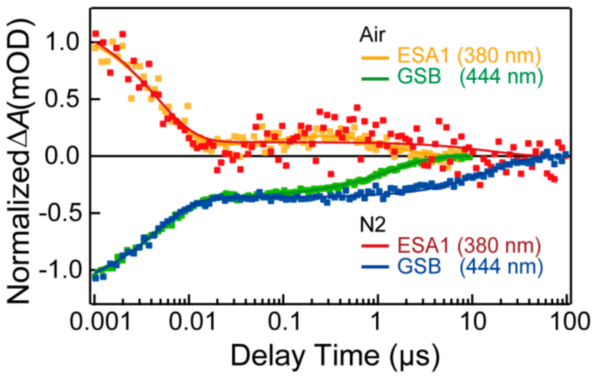

Figure S2. Comparison of proflavine lifetime before and after deoxygenation.

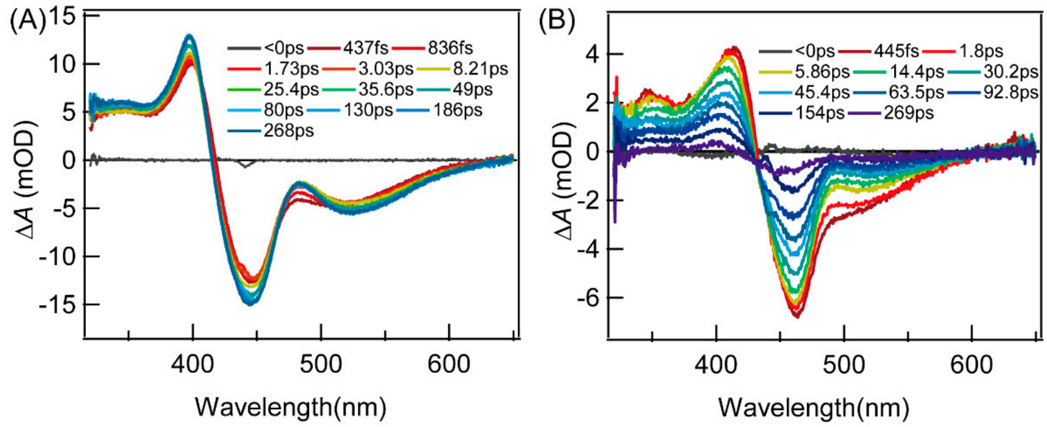

Figure S3. (**A**) Femtosecond time-resolved transient absorption spectra of the first 200 ps of proflavine (50 μM) in buffer solution (pH=7.4); (**B**) Femtosecond time-resolved transient absorption spectra of the first 200 ps of the intercalation complex of PF-DNA (50 μM) in buffer solution (pH=7.4).
